# Morphine Accumulates in the Retina Following Chronic Systemic Administration

**DOI:** 10.3390/ph15050527

**Published:** 2022-04-25

**Authors:** Nikolas Bergum, Casey-Tyler Berezin, Gregory Dooley, Jozsef Vigh

**Affiliations:** 1 Department of Biomedical Sciences, Colorado State University, Fort Collins, CO 80523, USA; nbergum@colostate.edu; 2 Cell and Molecular Biology Graduate Program, Colorado State University, Fort Collins, CO 80523, USA; ct.berezin@colostate.edu; 3 Department of Environmental and Radiological Health Sciences, Colorado State University, Fort Collins, CO 80523, USA; gregory.dooley@colostate.edu

**Keywords:** opioids, pharmacokinetics, morphine, retina, hypothalamus, mass spectrometry, blood-brain barrier, blood-retina barrier

## Abstract

Opioid transport into the central nervous system is crucial for the analgesic efficacy of opioid drugs. Thus, the pharmacokinetics of opioid analgesics such as morphine have been extensively studied in systemic circulation and the brain. While opioid metabolites are routinely detected in the vitreous fluid of the eye during postmortem toxicological analyses, the pharmacokinetics of morphine within the retina of the eye remains largely unexplored. In this study, we measured morphine in mouse retina following systemic exposure. We showed that morphine deposits and persists in the retina long after levels have dropped in the serum. Moreover, we found that morphine concentrations (ng/mg tissue) in the retina exceeded brain morphine concentrations at all time points tested. Perhaps most intriguingly, these data indicate that following chronic systemic exposure, morphine accumulates in the retina, but not in the brain or serum. These results suggest that morphine can accumulate in the retina following chronic use, which could contribute to the deleterious effects of chronic opioid use on both image-forming and non-image-forming visual functions.

## 1. Introduction

Opioids are some of the most effective analgesics and are therefore commonly used to treat both acute and chronic pain [1]. Despite their analgesic efficacy, opioid use has a plethora of negative, on-target side effects, including respiratory depression and gastrointestinal problems, as well as a high abuse liability [1,2,3]. Therefore, developing a better understanding of how opioids deposit in different tissues could elucidate the mechanisms underlying the sometimes-disparate effects of opioid drugs. Understanding drug pharmacokinetics is essential for optimizing treatment options and minimizing risk for patients [4].

Morphine is the prototypical opioid against which other opioid drugs are compared [2,5,6]. It can be administered via several different routes (oral, intravenous, intrathecal, etc.) for perioperative and postoperative pain management [5]. Morphine exerts its analgesic effects primarily via interactions with the µ-opioid receptor, with lesser contributions from its binding to the κ- and δ-opioid receptors [7]. Moreover, morphine is a major pharmacologically active metabolite of other opioids such as heroin and codeine [8,9,10,11,12]. Thus, studying the pharmacokinetics of morphine in different tissues provides information about the drug’s metabolism at different sites of action.

For morphine to exhibit its antinociceptive effect, it must access opioid receptors located in the central nervous system (CNS) by crossing the tightly regulated blood brain barrier (BBB) [13,14]. As a polar molecule, morphine trafficking across selective barriers such as the BBB is relatively poor. Thus, only a fraction of the morphine present in serum reaches the brain to affect the CNS neurons that mediate opioid-induced antinociception, locomotor activity, and respiratory depression [15,16,17,18]. Therefore, it is important to determine the amount of morphine that reaches the target neurons in the CNS to determine appropriate dosages.

Morphine’s uptake into the CNS is lower than other opioids due to its low lipid solubility [15]. Due to morphine’s hydrophilicity, it is thought that morphine transport across the BBB is primarily mediated by ATP-binding cassette (ABC) transporters and solute carrier (SLC) transporters [6,19]. Of these transporters, P-glycoprotein (P-gp) is the best studied in regard to its role in mediating morphine efflux from the CNS [6,14,19]. Indeed, genetic and pharmacological manipulations of P-gp expression/function show an inverse relationship between P-gp expression/function and the deposition of morphine in the brain [19,20,21,22,23]. The inner blood–retinal barrier (iBRB), the retinal analog of the BBB, expresses the same transporters found at the BBB, which are similarly thought to regulate drug transport between the retina and systemic circulation [24,25,26]. Interestingly, the relative expression of P-gp has been shown to be lower in the iBRB compared to the BBB [27]. However, whether reduced P-gp expression in the retina has functional consequences with regard to retinal morphine deposition remains unknown.

In forensic toxicology, the vitreous humor (VH) of the eye is often used to determine if an opioid overdose contributed to fatality [26,28,29,30,31,32]. Opioid drugs/metabolites in the VH are protected from postmortem degradation and redistribution, making it an ideal sample for postmortem toxicological investigations [26,28,29,32]. Since these VH samples are mostly collected postmortem from victims of opioid-related deaths, the pharmacokinetics of opioid drugs within the VH are relatively poorly understood compared to other tissues such as the brain and blood [9,11,28,29,31,33,34]. Thus, the mechanism that underlies the persistence of opioid drugs (and their metabolites) in the VH is unclear [26,35].

The present study examines the pharmacokinetics of morphine in the brain (hypothalamus) and retina/VH following systemic administration. Mice have been used extensively to study the analgesic and psychomotor effects of morphine (and other opioid drugs), as well as to examine the pharmacokinetics of opioids in the serum and brain [12,36,37,38]. Thus, we measured the morphine content of mouse retina/VH samples following intraperitoneal (i.p.) morphine injection(s) using liquid chromatography–tandem mass spectrometry (LC-MS/MS). Using this method, we were able to establish a pharmacokinetic profile for morphine in mouse retina/VH. Moreover, we measured the morphine content of the serum and hypothalamus samples from these same mice to directly compare the pharmacokinetics of morphine in the blood, brain, and retina/VH. Consistent with forensic toxicological findings, these experiments show that morphine persists in the retina/VH for up to 11 h following a systemic injection. Additionally, morphine can accumulate in the retina/VH upon repeat systemic exposure. Significantly, morphine levels in the retina/VH exceed the concentrations found in the brain at all time points. Indeed, the retina shows reduced expression of the morphine extruder P-gp compared to the hypothalamus, which could explain the persistent high morphine levels in the retina following systemic exposure. Given the evidence that some retinal neurons express opioid receptors [39,40], the accumulation of morphine in the retina could result in changes in image-forming, as well as non-image-forming, visual functions. Thus far, no visual deficits have been reported that would directly implicate opioid-induced interference with image-forming visual circuitry. However, pupillary light reflex (PLR) is altered in opioid-tolerant patients [41]. Similarly, mice intraocularly injected with the MOR-selective agonist [D-Ala2, N-MePhe4, Gly-ol]-enkephalin (DAMGO) exhibit deficits in PLR [42]. These observations support the idea that morphine accumulation in the retina could have functional consequences within the eye.

## 2. Results

### 2.1. Morphine Deposits in Mouse Retina Following Systemic Exposure

**Figure 1:**
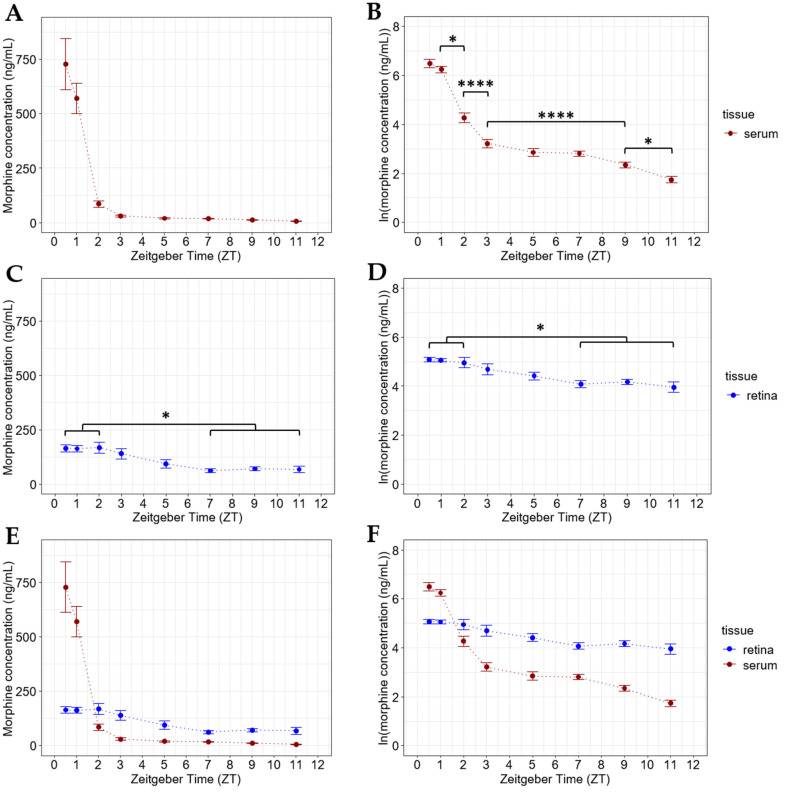
Morphine deposits in the retina following systemic exposure. (**A**) Serum morphine concentrations and (**B**) natural logarithmic (ln) transformation of serum morphine concentrations collected from mice sacrificed at specified time points following a 20 mg/kg i.p. morphine injection at light onset (ZT 0). One-way ANOVA with a Tukey post hoc adjustment was performed on an ln scale to compare serum concentrations from animals sacrificed at different time points (* = *p* < 0.05, **** = *p* < 0.0001). (**C**) Retinal morphine concentration and (**D**) ln transformation of retinal morphine collected from the same mice (as in **A** and **B**) sacrificed at specified time points following a 20 mg/kg i.p. morphine injection at light onset (ZT 0). One-way ANOVA with a Tukey post hoc adjustment was performed on an ln scale (* = *p* < 0.05). (**E**) Serum and retinal morphine concentrations and (**F**) ln transform of serum and retinal morphine concentrations collected from mice sacrificed at specified time points following a 20 mg/kg i.p. morphine injection at light onset (ZT 0). Retina and serum samples were collected from the same mice (as in **A**–**D**) within each measured time point. (**F**) Significant differences exist within each plotted time point between the retinal and serum concentrations (*p* < 0.001). Two-way ANOVA with a tissue *x* ZT interaction and a Tukey post hoc adjustment was performed on an ln scale. Data are presented as the mean ± SEM, with 8–16 mice per group.

To examine the pharmacokinetics of morphine in mouse retina, we injected mice with 20 mg/kg morphine i.p. at light onset (Zeitgeber Time (ZT) 0). Whole retina and serum samples were then collected from mice sacrificed at 0.5 (*n* = 8), 1 (*n* = 14), 2 (*n* = 11), 3 (*n* = 14), 5 (*n* = 10), 7 (*n* = 8), 9 (*n* = 16), and 11 (*n* = 13) hours following the i.p. injection. The morphine content of these samples was measured using LC-MS/MS. Serum morphine peaked around 0.5–1 h post-injection, before dropping to low levels (<50 ng/mL) after just 3 h (Figure 1A,B). These findings are consistent with past studies that examined the serum pharmacokinetics of morphine in mice [12,37,38]. Importantly, while morphine levels peaked at 0.5–1 h post-injection, serum morphine levels were lowest (3.3 ng/mL) 11 h following the injection (Figure 1B).

From these same mice, we collected whole retina/VH samples and measured their morphine content using LC-MS/MS. Given the proximity of the VH to the retina, as well as the small volume of the mouse VH, we were unable to reliably separate the VH from the retina. Thus, the retina samples tested contained both retina and VH. Henceforth, when referring to retina, this represents samples that contain both retina and VH. Interestingly, we detected morphine in the retina of these mice 0.5–11 h following the 20 mg/kg i.p. injection at ZT 0 (Figure 1C,D). In contrast to the serum morphine levels from these animals, the retinal morphine levels were highest between 0.5–2 h following the injection before slowly dropping and stabilizing around 70 ng/mL (Figure 1C,D). When comparing the serum and retina morphine pharmacokinetics (from samples collected from the same animals), we observed that the serum morphine exceeded the retinal morphine concentration 0.5–1 h following the injection. However, the serum morphine levels soon dropped and remained lower than the morphine concentration in the retina for the remainder of the detection period (Figure 1E). There were statistically significant differences (*p* < 0.001) between the serum and retinal morphine concentrations at each time point (Figure 1F). Perhaps the most striking finding is that morphine persists in the retina at around 70 ng/mL long after it has dropped to <10 ng/mL in the serum (Figure 1E,F).

### 2.2. Morphine Accumulates in Mouse Retina Following Repeated Systemic Exposure

**Figure 2:**
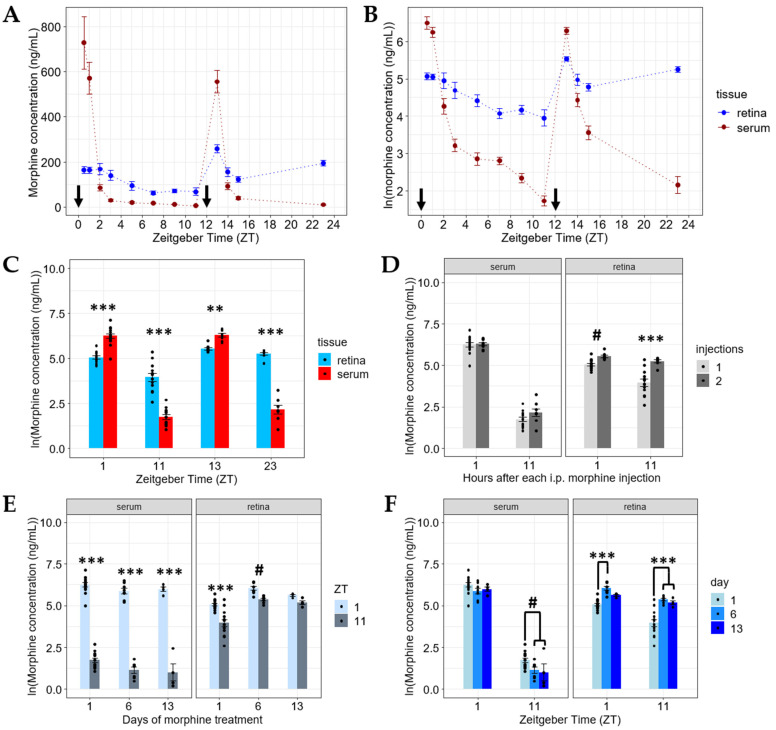
Morphine accumulates in the retina following repeated systemic exposure. (**A**) Morphine pharmacokinetics in retina and serum following 20 mg/kg i.p. morphine injections at ZT 0 and ZT 12 (indicated by arrows). (**B**) Morphine concentrations in retina and serum following systemic injections at ZT 0 and ZT 12 (indicated by arrows) on the natural logarithmic (ln) scale. Significant differences exist between the retina and serum within each measured time point (*p* < 0.01). Two-way ANOVA was used with a Tukey post hoc adjustment for the ln scale. (**C**) Morphine concentration in the serum is higher than in the retina 1 h (ZT 1 and ZT 13) after 20 mg/kg i.p. morphine injections at ZT 0 and ZT 12 (at the selected time points from the data shown in **B**). However, the retinal morphine levels exceed the serum levels at 11 h after systemic exposure (ZT 11 and ZT 23). Two-way ANOVA with a tissue *x* ZT interaction and a Tukey post hoc adjustment was performed on the ln scale. (**D**) Serum morphine pharmacokinetics remain the same at 1 and 11 h after one and two 20 mg/kg i.p. morphine injections, while the retinal morphine levels increased following the second injection at those same time points. Three-way ANOVA with a tissue *x* hours post-injection *x* injection number interaction and a Tukey post hoc adjustment was performed on the ln scale. (**E**,**F**) Morphine appears to accumulate in the retina following 6 or 13 days of chronic systemic morphine treatment, while the serum pharmacokinetics remain similar for ZT 1 and ZT 11. Three-way ANOVA with a tissue *x* ZT *x* day interaction using a Tukey post hoc adjustment was performed on the ln scale. (# = *p* < 0.05, ** = *p* < 0.001, *** = *p* < 0.0001). Data are presented as the mean ± SEM.

After establishing the pharmacokinetics of morphine in the retina relative to serum following a single systemic injection, we wanted to assess the pharmacokinetics of morphine in these tissues following a subsequent, as well as after chronic, i.p. morphine injections. To assess this, we first collected serum and whole retina samples at various time points (ZT 13: *n* = 9, ZT 14: *n* = 8, ZT 15: *n* = 8, and ZT 23: *n* = 8) from mice treated with 20 mg/kg morphine i.p. at both light onset (ZT 0) and light offset (ZT 12). Like the single injection data, the serum morphine levels exceeded the retinal morphine concentrations an hour (ZT 13) after the second i.p. injection (Figure 2A–C). Additionally, the serum morphine dropped significantly below the retinal morphine concentrations 2 h (ZT 14) after the second injection (Figure 2C) and remained below the retina morphine levels for the remainder of the detection period (Figure 2A–C). Interestingly, when comparing retinal morphine levels both 1 (ZT 13) and 11 (ZT 23) hours after the second morphine injection, we saw an increase (*p* < 0.05) compared to the retinal morphine levels from samples collected 1 (ZT 11) and 11 (ZT 23) hours after just a single i.p. morphine injection (Figure 2D). Contrastingly, there were no significant differences (1 h: *p* = 0.8238, 11 h: *p* = 0.547) detected in the serum morphine levels at these same time points when comparing one versus two morphine injections (Figure 2D). Next, we wanted to assess how chronic morphine treatment would affect the deposition of morphine in the retina. The chronic morphine paradigm consisted of twice daily 20 mg/kg i.p. morphine injections (at ZT 0 and ZT 12) for 5 or 12 days [43]. On day 6 or day 13, retina and serum samples were collected from these mice at the specified time points after a final 20 mg/kg i.p. injection at ZT 0. Morphine measurements from these mice revealed increases in retinal morphine concentrations (at all comparable time points) between the mice treated with just a single morphine injection (day 1) and mice chronically treated with morphine (day 6 and day 13) (Figure 2F and Figure S1B). Moreover, the difference in retinal morphine between 1–2 h (Figure 2F: Day 6 ZT 1: *n* = 8, Day 13 ZT 1: *n* = 4; Figure S1B: Day 13 ZT 2: *n* = 4) and 9–11 h (Figure 2F: Day 6 ZT 11: *n* = 7, Day 13 ZT 11: *n* = 4; Figure S1B: Day 13 ZT 9: *n* = 4) disappeared after 13 days of chronic morphine exposure, while the serum pharmacokinetics remained similar (Figure 2E and Figure S1B). Intriguingly, the chronic-morphine-treated animals (day 6 & day 13) showed a modest, yet significant decrease (*p* < 0.05) in serum morphine concentration both 9 (ZT 9) and 11 (ZT 11) hours after the final morphine injection (when compared to day 1 levels) (Figure 2F and Figure S1B).

### 2.3. Morphine Concentration in Mouse Retina Exceeds the Hypothalamic Morphine Concentration Following Systemic Exposure

**Figure 3:**
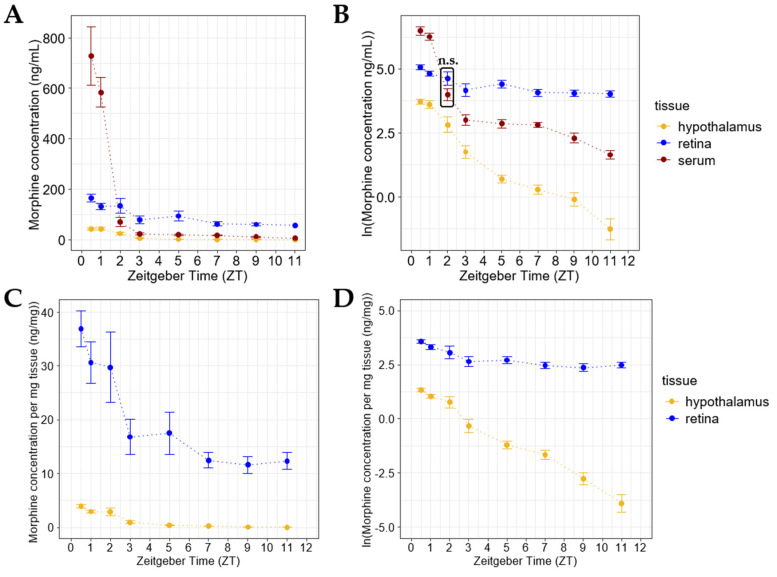
Morphine deposits more in the retina than in the hypothalamus following systemic administration. (**A**) Retinal morphine concentrations exceed hypothalamic morphine concentrations at all measured time points following a single i.p. morphine injection. (**B**) Morphine concentrations between the hypothalamus, retina, and serum were all significantly different at each measured time point following a single i.p. morphine injection (*p* < 0.001), except for comparison shown in the box at ZT 2 (n.s. = not significantly different). Two-way ANOVA with a Tukey post hoc adjustment was performed on a natural logarithmic (ln) scale (**C**,**D**). Morphine concentration in the retina exceeds that detected in the hypothalamus when normalized against tissue weight at each measured time point following a single i.p. morphine injection (*p* < 0.0001). (**D**) Two-way ANOVA with a Tukey post hoc adjustment was performed on an ln scale. Data are presented as the mean ± SEM, with 6–10 mice per group.

Since retinal morphine levels seem to increase upon repeated systemic exposure, we wanted to compare the deposition of morphine in the retina to the brain. We chose to compare the retina to the hypothalamus, as the hypothalamus has been shown to have the highest concentration of morphine following systemic exposure (when compared to other brain regions) [44]. Using solid phase extraction followed by LC-MS/MS, we were able to reliably measure the morphine content of brain tissue, including the hypothalamus and cortex. Despite the hypothalamus’ proximity to the median eminence, we did not detect any differences in morphine deposition between the hypothalamus and the cortex (Figure S2). Interestingly, the hypothalamic morphine concentration was lower than both the retinal and serum morphine levels at 0.5 (*n* = 8), 1 (*n* = 17), 2 (*n* = 10), 3 (*n* = 7), 5 (*n* = 10), 7 (*n* = 8), 9 (*n* = 8), and 11 (*n* = 6) hours after a single i.p morphine injection at ZT 0 (Figure 3A,B). When the morphine concentrations of the retina and hypothalamus were normalized to their weights, the retinal morphine concentrations still vastly exceeded the morphine levels detected in the hypothalamus (Figure 3C,D). Additionally, the retinal morphine concentration per tissue weight remained much higher (*p* < 0.0001) than the hypothalamic morphine concentration in samples collected 1, 2, and 3 h after a second i.p. morphine injection at ZT 12 (Figure S3).

### 2.4. Morphine Accumulates in Mouse Retina but Not in the Hypothalamus

**Figure 4:**
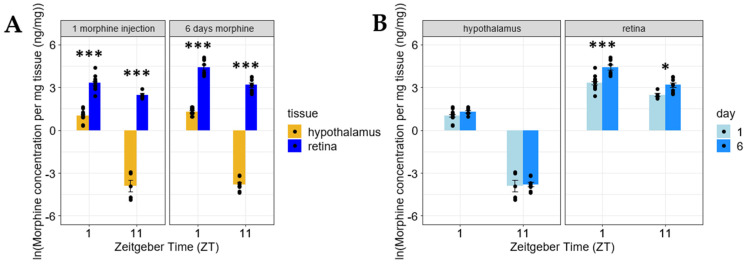
Morphine accumulates in the retina, but not the hypothalamus. (**A**) Retinal morphine concentrations exceed hypothalamic morphine concentrations at 1 (ZT 1) and 11 (ZT 11) hours following chronic morphine exposure (6 days of morphine treatment). (**B**) Morphine accumulates in the retina, but not the hypothalamus following chronic morphine exposure. Two-way ANOVA with a Tukey post hoc adjustment was performed on a natural logarithmic (ln) scale. Data are presented as the mean ± SEM with 5–17 mice per group (* = *p* < 0.01, *** = *p* < 0.0001).

After observing the significant difference in morphine penetrance within two CNS tissues (retina and brain) after a single systemic injection, we measured the levels of morphine in the retina and hypothalamus after 6 days of chronic morphine treatment. The retinal morphine content exceeded the morphine concentrations in the hypothalamus; futhermore, chronic morphine exposure appeared to exacerbate the differences in morphine concentrations between these tissues (Figure 4A). These analyses revealed that chronic morphine administration preserved the differences in morphine concentrations between the retina and hypothalamus at both 1 (Day 6 ZT 1: *n* = 8) and 11 (Day 6 ZT 11: *n* = 8) h after the ZT 0 systemic injection. Intriguingly, morphine seems to accumulate in the retina following chronic systemic exposure, while morphine levels in hypothalamus appear to remain the same (Figure 4B). Figure S4B even shows that at ZT9 following chronic morphine exposure, hypothalamic morphine levels drop following 13 days of chronic morphine, while the retinal morphine concentration is significantly larger (*p* < 0.01) than the single injection levels following chronic administration.

### 2.5. Reduced P-gp Expression in the Retina May Underlie Retinal Morphine Accumulation

**Figure 5:**
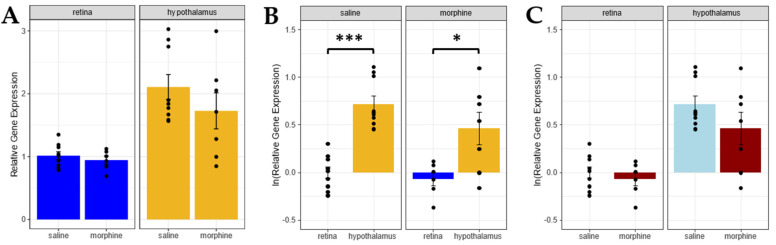
Reduced P-gp expression in the retina may underlie retinal morphine accumulation. P-pg mRNA expression (**A**) and natural logarithm (ln) (**B**) of P-gp mRNA expression is lower in the retina than in the hypothalamus at one hour (ZT 1) after an i.p. morphine injection at ZT 0. (**C**) Chronic morphine exposure does not affect P-gp mRNA expression in either tissue depositions at ZT 1. Two-way ANOVA with a Tukey post hoc adjustment was performed on an ln scale. Data are presented as the mean ± SD (* = *p* < 0.01, *** = *p* < 0.0001).

P-gp’s role in transporting morphine from the brain into systemic circulation is well established [19,20,21,22,23]. Significantly, this important transporter is also expressed in the iBRB, albeit at lower levels than in the brain [27,45]. Thus, we wanted to assess the role of P-gp within the context of differential morphine deposition in the retina versus the hypothalamus following systemic exposure. To investigate the role of P-gp in morphine transport within these different CNS tissues, we injected mice with 20 mg/kg morphine (*n* = 7) or saline (*n* = 9) i.p. at ZT 0 and ZT 12 for 6 days. Retina and hypothalamus samples were collected from these mice and total RNA from these tissues was extracted for quantitative reverse-transcription PCR (qRT-PCR) analysis. The expression of *Abcb1a* (gene transcript coding for P-gp) mRNA, relative to the reference genes B-actin and Tbp, was calculated using the saline retina samples as the control. Relative gene expression analyses revealed that *Abcb1a*/P-gp mRNA expression in the retina was lower than the expression in the hypothalamus (Figure 5A,B). Importantly, chronic morphine exposure did not alter the expression of *Abcb1a*/P-gp mRNA in the brain (hypothalamus) or retina (Figure 5C).

## 3. Discussion

The data presented provide an in depth look at morphine pharmacokinetics in the retina, as well as in the serum and hypothalamus, for longer durations than previously reported. Consistent with past findings, these data suggest that morphine levels in the serum peak within the first hour after i.p. administration, and then drop rapidly to low levels within 1–2 h following the injection [37,38,46]. While the serum pharmacokinetics of past studies focused on the serum morphine levels within the first 3 h after injection, this study measured serum morphine up to 11 h following injection. Analysis of this data showed consistent decreases in serum morphine content up to 11 h after the initial injection.

As previously mentioned, opioid metabolites are routinely detected from postmortem human VH samples; however, these samples are often collected from overdose victims following the administration of a fatal opioid dose [26,28,29,30,31,32]. While it is unclear whether sub-lethal opioid doses similarly accumulate in human VH/retina, animal studies suggest that sub-lethal doses of opioids accumulate in the mammalian VH/retina following systemic exposure [11,31,47]. While the current study differs in species, treatment, and methodology, it builds on this past work by providing an examination of morphine pharmacokinetics within mouse retina following both acute and repeated systemic morphine administration.

To our knowledge, no study has measured retinal morphine levels in mice following systemic exposure. Therefore, we developed and validated a method for detecting morphine levels within the retina/VH samples of systemically morphine-treated mice. The data from these experiments indicate that morphine indeed deposits in the retina following systemic exposure and even persists at elevated levels relative to the serum and hypothalamus. These findings are consistent with the idea that opioid metabolites in the VH are resistant to postmortem redistribution, and thus have an extended detection window within the VH/retina compared to other tissues [11,26]. In humans, morphine is metabolized into morphine-3 glucuronide (M3G) and morphine-6 glucuronide (M6G) via UDP-glucuronyl-transferase (UGT) [7,48,49]. Of these metabolites, M3G does not contribute to morphine’s analgesic effect [49,50,51] or to morphine-induced behavioral activation [37], while M6G does [52]. However, mice produce little to no M6G following morphine administration [12,37,53].

Importantly, following a second systemic injection, the morphine levels in the retina seem to accumulate, while the pharmacokinetics of serum morphine in the same animals remain relatively constant when compared 1 and 11 h after one or two injection(s). This retinal accumulation following repeated systemic exposure is further evidenced when comparing retina samples collected at ZT 1 and ZT 11 after a single injection to samples collected at ZT 1 and ZT 11 from animals following chronic systemic morphine exposure. Interestingly, the levels of morphine in the serum after 11 h (ZT 11) appear to be even lower in animals chronically treated with morphine than after a single i.p. morphine injection. While few other studies have examined the long-term serum pharmacokinetics of morphine, we suspect that this may be due metabolic adaptations (i.e., increased UGT2B activity) that mice undergo in response to a chronic morphine paradigm [7,54].

Morphine deposition and transport into the brain has been extensively studied as it is necessary for the analgesic efficacy of morphine [13,14]. Since mice are used extensively as a mammalian model for opioid-related biomedical research, the pharmacokinetics of morphine and other opioids in the mouse brain has been well characterized [12,38,55,56,57,58]. Although we initially suspected that the hypothalamus might have increased morphine penetrance due to its proximity the median eminence [44,59], we found no difference in morphine concentrations compared to cortices harvested from the same animals (Figure S1). With that being said, the data we obtained regarding the pharmacokinetics of morphine in the brain were consistent with past findings, with peak concentrations of hypothalamic morphine levels detected around 30 min to 1 h following the time of injection [12,38]. Nonetheless, this study offers a more in depth look at the brain pharmacokinetics of morphine up to 11 h following systemic administration. In many ways, the pharmacokinetics of morphine in the hypothalamus mirror that of the serum, as both serum and brain morphine concentrations peak about an hour after systemic exposure and continuously drop over time. Contrastingly, the retinal morphine concentration remains high compared to the serum and hypothalamus. Perhaps most significantly, our data suggest that while the morphine appears to be accumulating in the retina/VH following chronic systemic exposure, the concentrations in the brain do not. Both CNS tissues have structurally similar barriers (the iBRB and BBB) that selectively regulate the transport of xenobiotics. Past experiments have shown that the genetic and pharmacological manipulation of P-gp alter morphine concentration within the brain [19,20,21,22,23]. In fact, experimental evidence suggests that P-gp expression and brain morphine levels have an inverse relationship, as increased P-gp expression results in lower levels of morphine within the brains of mice [22]. Our data are consistent with this idea, as the relative P-gp mRNA (*Abcb1a*) expression within the retina is significantly lower than expression levels in the hypothalamus.

Initially, it was believed that chronic morphine exposure could alter the expression of P-gp mRNA at the BBB [60,61]. However, it was later observed that the cessation of chronic morphine treatment, rather than the chronic morphine treatment itself, likely resulted an increase in P-gp mRNA [62]. Our data support the latter of the two findings, as our chronic morphine treatment did not alter the P-gp mRNA in with either tissue. Future studies shall investigate the role of other BBB transporters (such as Breast Cancer Resistance Protein and Multidrug Resistance Protein 2) to assess their potential roles in the pharmacokinetics of morphine within both the brain and the retina.

Because the analgesic and psychomotor effects of morphine are heavily dependent on the concentration within the brain and spinal cord, the opioid pharmacokinetics within these regions have been extensively studied [13,14,37,38]. Contrastingly, the retina/VH has been less studied in regard to opioid pharmacokinetics as it has been primarily employed as a sample for postmortem toxicological investigations. This study presents an in-depth pharmacokinetic characterization of morphine in the retina relative to serum and hypothalamus, highlighting that morphine persists and accumulates in the retina following chronic systemic exposure. Since no visual phenotype has been reported following chronic opioid exposure, it was previously assumed that systemic opioid exposure did not affect structures in the eye. However, chronic morphine exposure was shown to promote neovascularization in the retina, which could contribute to retinopathy in patients with sickle cell disease [63]. Although opioids often have profound deleterious effects, the administration of chronic systemic morphine protected against hypertension-mediated retinal ganglion cell death in rats [47]. Additionally, rabbit studies have indicated that retinal morphine might reduce ischemia-induced retinal ganglion cell death following systemic exposure [64,65]. As these previous studies suggest, opioids may affect retinal ganglion cells through opioid receptors expressed on these cells [39]. Interestingly, our group has identified a subset of intrinsically photosensitive retinal ganglion cells (ipRGCs) that express the µ-opioid receptors (MOR), which are the main molecular targets for opioid drugs such as morphine [40]. Moreover, these studies have shown that the MOR-selective agonist [D-Ala2, N-MePhe4, Gly-ol]-enkephalin (DAMGO) not only directly affects ipRGC firing activity via the modulation of specific ionic currents, but also that MOR activation attenuates the ipRGC-dependent PLR in mice [40,42].

Perhaps most importantly, ipRGCs are crucial for the synchronization of sleep/circadian behavior to environmental light–dark cycles through a process known as photoentrainment [66]. These ipRGCs send environmental light information to key sleep and circadian centers located in the hypothalamus such as the suprachiasmatic nucleus (SCN) and the ventrolateral preoptic area (VLPO) [66]. Moreover, the opioid fentanyl has been shown to alter photoentrainment in hamsters, presumably though alteration of electrical activity and clock gene expression within the SCN [67,68,69]. Additionally, opioid-induced modulation of the VLPO increases wakefulness in rodents via MORs within this region [70,71].

Given this evidence, it is believed that chronic opioid-related sleep/wake disturbances are due to opioid-induced alterations to key sleep centers within the brain [72,73], while the retinal contributions to opioid-mediated sleep disruption are seldom considered. However, retinal morphine levels appear to exceed the EC_50_ for the inhibition of calcium current (an established effector of ipRGC MORs) after repeated systemic morphine exposure [74]. This suggests that chronic systemic morphine exposure within these mice might activate MORs present on the ipRGCs, thus affecting their ability to transmit important environmental light information to brain’s sleep/wake centers. Taken together, the present data point to an intriguing possibility that retinal morphine accumulation might contribute to chronic opioid-induced sleep/wake disturbances.

## 4. Materials and Methods

### 4.1. Animals

All animals used in these studies were handled in compliance with the Institutional Animal Care and Use Committees of Colorado State University (Protocol 18-8395A, 28 January 2019) and in accordance with the ARVO Statement for the Use of Animals in Ophthalmic and Vision Research. The animals were housed under a 12:12 light/dark (LD) cycle, with lights on at 7:00 a.m. (ZT 0) and lights off at 7:00 p.m. (ZT 12). Food and water were made available ad libitum.

### 4.2. Morphine Treatment

Mice were weighed and injected with a single i.p. dose of 20 mg/kg morphine (Morphine sulfate salt pentahydrate, Sigma-Aldrich Saint Louis, MO, USA; Product Number: M8777, dissolved in sterile saline) at light onset (ZT 0). The dosage of 20 mg/kg i.p. morphine is commonly used to assess analgesic tolerance in mice [75]. Mice that received two injections received a second 20 mg/kg i.p. morphine injection at light offset (ZT 12). The chronic morphine treatment paradigm consisted of 5 or 12 days of twice daily 20 mg/kg morphine (administered at ZT 0 and ZT 12), followed by a final 20 mg/kg i.p. injection at ZT 0 on day 6 or 13 of the treatment paradigm [43].

### 4.3. Tissue Sample Collection: Retina, Hypothalamus, and Serum

Mice were deeply anesthetized with isoflurane, decapitated, and trunk blood was collected into EDTA-coated tubes (BD Vacutainer K2 EDTA 7.2 mg tube 4.0 mL). Then, the hypothalamus was removed, weighed, and placed in tubes containing 100 µL 0.1 M PBS. Whole retinas were then microdissected from the eyes of each animal and placed in tubes containing 100 µL 0.1 M PBS. Notably, the retina samples tested contained both retinal tissue and VH.

### 4.4. Morphine Analysis by LC-MS/MS

#### 4.4.1. Retina Batch Preparation

To prepare retina samples for analysis, 10 µL of 10 µg/mL D6-morphine internal standard solution (Morphine-D6 solution, Cerilliant, Round Rock, TX, USA) was added to whole-retina samples and matrix-matched standards. Here, matrix-matched standards were whole-retinal samples collected from untreated mice spiked with a morphine standard (Morphine solution, Sigma-Aldrich Saint Louis, MO, USA; Product Number: M-005). Subsequently, 200 µL ice-cold acetonitrile was added to the retina samples/standards and the samples/standards were sonicated at maximum power (40 kHz) for 30 min using a Branson Bransonic^®^ (Emerson Electric Co., Saint Louis, MO, USA) 5800 Ultrasonic bath (with bath water at room temperature). Retina samples were then spun down at 14,000 rpm for 5 min, and 100 µL supernatant was added to sample vials containing 200 µL 0.1% formic acid in water and then transferred to autosampler vials fitted with 400 μL glass inserts. The limit of detection was 20 ng/mL morphine.

#### 4.4.2. Hypothalamus Batch Preparation

To prepare hypothalamus samples for analysis, 10 µL of 1 µg/mL D6-morphine in-ternal standard solution was added to hypothalamus samples and matrix-matched standards. Here, matrix-matched standards were hypothalamus samples collected from untreated mice spiked with a morphine standard. Hypothalamus samples/standards were then homogenized for 10 s using a BeadBug™ (Benchmark Scientific, Inc. Sayreville, NJ, USA) 3 Position Bead Microtube homogenizer in 400 µL ice-cold acetonitrile. Samples were then centrifuged 14,000 rpm for 5 min and supernatant was added to Phenomenex Strata-X-Drug B solid phase columns. Columns were washed with 2 mL 0.1% formic acid in water and 2 mL methanol. Columns were then dried for 10 min prior to elution. Two successive aliquots of a solution containing 50% acetonitrile, 42% methanol, and 8% 7N ammonium in methanol were used to elute samples from the columns. Eluents were collected into a clean glass test tube and dried under nitrogen at 40 °C. Dried eluents were reconstituted with 200 µL 95:5% water:acetonitrile and transferred to autosampler vials fitted with 400 μL glass inserts. The limit of detection was 0.2 ng/mL morphine.

#### 4.4.3. Serum Batch Preparation

To prepare serum samples for analysis, 10 µL of 1 µg/mL D6-morphine internal standard solution was added to serum samples and matrix-matched standards. Here, matrix-matched standards were serum samples collected from untreated mice spiked with a morphine standard. Serum samples/standards were prepared for solid phase extraction by adding 20 µL zinc sulfate (5% weight/volume), followed by the addition of 300 µL ice-cold acetonitrile to each tube. Samples were then centrifuged 14,000 rpm for 5 min and supernatant was added to Phenomenex Strata-X-Drug B solid phase columns. Columns were washed with 2 mL 0.1% formic acid in water and 2 mL methanol. Columns were then dried for 10 min prior to elution. Two successive aliquots of a solution containing 50% acetonitrile, 42% methanol, and 8% 7N ammonium in methanol were used to elute samples from the columns. Eluents were collected into a clean glass test tube and dried under nitrogen at 40 °C. Dried eluents were reconstituted with 200 µL 95:5% water: acetonitrile and transferred to autosampler vials fitted with 400 μL glass inserts. The limit of detection was 0.5 ng/mL morphine.

#### 4.4.4. Data Acquisition and Analysis

Samples were analyzed with an Agilent 1290 UHPLC coupled to an Agilent 6460 triple quadruple mass spectrometer equipped with an Agilent Jet Stream electrospray ionization. Morphine was chromagraphically separated on a Restek Raptor biphenyl column (3.0 × 50 mm, 2.7 μm) held at 40 °C. A sample volume of 10 μL was injected into a mobile phase mixture of 95% water with 0.1% formic acid (A) and acetonitrile with 0.1% formic acid (B). The flow rate of 0.4 mL/min was kept consistent throughout the run time. The mobile phase composition was held at 3% B for 1.5 min, increased to 40% B at 3 min, and finished at 100% B at 4 min. The ionization source conditions used were as follows: positive polarity and nebulizer 40 psi; gas flow of 10 L/min at 320 °C; sheath gas flow of 12 L/min at 380 °C; capillary voltage of 3750 V; and nozzle voltage of 500 V. The ion transitions monitored were 286.2 → 152 and 128 *m*/*z* for morphine and 292.2 → 152 and 128 *m*/*z* for D6-morphine. Analytes were confirmed by retention time and the product ion ratio correlation between the sample peaks and corresponding standards (±20%). The data collection and processing were performed by using Agilent (Santa Clara, CA, USA) Mass Hunter Quantitative software (v.B.08.01). Quantitation was performed with linear regression using seven-point calibration curves from 0.5 ng/mL to 500 ng/mL (for hypothalamus and serum) or six-point calibration curves from 5 ng/mL to 1 µg/mL (for retina). These calibration curves were made from matrix (hypothalamus, serum, or retina tissue derived from untreated animals) spiked with morphine standards (morphine solution, Sigma-Aldrich, Saint Louis, MO, USA; Product Number: M-005).

#### 4.4.5. Method Validation

Prior to assessing the morphine concentrations of tissues derived from the morphine-treated animals, LC-MS/MS methods were validated for all three matrices (retina, serum, and hypothalamus). Matrices were validated using the following parameters: accuracy, precision, calibration model, carryover, interference, limit of quantitation (LOQ), and sample stability (see Tables S2 and S3 for validation results and acceptance criteria).

### 4.5. qRT-PCR

#### 4.5.1. RNA Preparation

Male WT mice received morphine or saline as described above. One hour after the injection (ZT 1), the mice were anesthetized with isoflurane and sacrificed by decapitation. Hypothalamic tissue was dissected from the brain and immediately homogenized for RNA extraction, while retinas were microdissected in 0.1 M phosphate buffered saline (PBS; pH 7.4) and placed in RNAlater solution (Sigma-Aldrich R0901) at 4 °C until after hypothalamic RNA was extracted. Total RNA was extracted from both tissues using the RNeasy Mini Kit (Qiagen, Hilden, Germany) according to the manufacturer’s instructions. Briefly, the tissues were lysed in Buffer RLT with DTT (GoldBio, St. Louis, MO, USA) by disruption with a micropipette, followed by homogenization of the solution by passing it through a 20 gauge needle five times. The lysate was centrifuged, and the supernatant was combined with 70% ethanol before being put on the column. After RNA was eluted from the column, the concentration and quality (A260/A280) of each sample was assessed using a Nanodrop (ThermoFisher NanoDrop Lite, Waltham, MA, USA). Genomic DNA (gDNA) was digested using DNase I and RNase-free (ThermoFisher) according to the manufacturer’s instructions. After DNase treatment, the RNA concentration was again measured using a Nanodrop, and RNA integrity and successful DNase treatment was assessed on a 1% agarose gel. All samples displayed strong 28S and 18S rRNA bands, no gDNA contamination, and no degradation.

#### 4.5.2. Reverse Transcription

Reverse transcription of RNA was performed using the GoScript™ Reverse Tran-scription System (ProMega, Madison, WI, USA) according to the manufacturer’s instructions. In each rotation, 200 ng RNA was used and a no-reverse-transcription (NRT) control was performed alongside each sample. cDNA was stored at −20 °C until it was used for qPCR amplification.

#### 4.5.3. qRT-PCR Primer Design

Previously published primers against *Abcb1a* (the rodent gene for p-glycoprotein [p-gp]) in rats [58] were adapted for use for the mice. The probe sequence for *Abcb1a*, as well as primer and probe sequences for the reference genes *β-actin* and *Tbp*, were designed using Integrated DNA Technologies’ PrimerQuest™ Tool. *β-actin* is a commonly used reference gene that has been used successfully in mouse retina [76], and *Tbp* has been shown to be an appropriate reference gene for adult mouse retina [77].

For each target, primers were designed to span an exon–exon junction, have melting temperatures of between 60 °C and 65 °C, have a GC content of between 45–65%, and be no more than 30 nucleotides long. Probes were designed to have melting temperatures at least 5 °C higher than the primers, have a GC content of between 45–75%, and be no more than 30 nucleotides long. Amplicons were required to be shorter than 150 nucleotides. All other parameters were left as defaults. The multiplexed primers (500 nM) and probe (250 nM) were resuspended in an IDTE buffer (10 mM Tris, 0.1 mM EDTA in H_2_O) upon receipt, then stored at −20 °C. The primer and probe sequences can be found in Table S1.

PCR products from each primer set were run on a 2% agarose gel to assess the specificity of the primers. For all genes, one clear band was seen with no gDNA contamination or additional products. The reaction parameters were first determined in singleplex reactions before multiplexing. For each primer set, a temperature gradient was used to determine the optimal annealing temperature.

#### 4.5.4. qRT-PCR Protocol

Reactions were set up using GoTaq^®^ Probe qPCR Master Mix (ProMega) according to the manufacturer’s instructions; however, because our primers and probes arrived multiplexed, only 1 µL total was used, and an additional 2 µL of nuclease-free water was added to the total 20 µL reaction mix. The cycling conditions were as follows: 2 min, 95 °C, GoTaq^®^ DNA polymerase activation, and then 35 cycles of denaturation (95 °C, 15 s) and annealing/extension (63 °C, 30 s).

Each plate contained a standard curve run in triplicate, which included three 10-fold dilutions. Every experimental sample was run in triplicate using 2 µL of cDNA. Both reference genes were stably expressed in all samples. A NRT control and no-template control (H_2_O instead of cDNA or RNA; NTC) was included in each run. No amplification was seen in any of the NRT or NTC controls. Assay plates were prepared in-lab, then run on a CFX96 Touch Real-Time PCR Detection System (BioRad, Hercules, CA, USA).

#### 4.5.5. Data Analysis

The CFX Manager™ Software Version 3.1 (BioRad, Hercules, CA, USA) was used to set the threshold for each reaction (the highest R2 just above the background) and to assess the efficiency of the reaction based on the standard curve (90–110%). It was also used to normalize the data between plates when samples needed to be re-run or run on multiple plates. In these cases, at least one identical sample was run on each plate and used as an inter-run calibrator. The Cq values for each experiment were downloaded as an Excel spreadsheet and the ΔΔCt method was used for analysis [78,79]. For each gene, the Cq values for all samples in the control group were averaged and used as the reference to calculate the relative gene expression (RGE) in each sample. Because multiple reference genes were used, the geometric mean of those genes’ relative quantities was used to calculate the RGE [80].

### 4.6. Data Collection and Statistical Analysis

Data are presented as mean ± standard error of the mean (SEM) or mean ± standard deviation (SD), as specified in figure captions. The data were analyzed using One-way, two-way, or three-way ANOVA on a natural logarithmic scale to satisfy ANOVA assumptions. Data visualizations and analyses were performed using RStudio (version 4.0.0) with Tukey’s post hoc adjustments for all pairwise comparisons, and *p* < 0.05 was considered significant.

## 5. Conclusions

In this study, we measured morphine concentrations in mouse retina. The data reveal that morphine deposits and accumulates in mouse retina following both acute and chronic systemic exposure. Importantly, morphine concentrations in the retina accumulate following chronic systemic administration and vastly exceed brain morphine concentrations across all sampled time points. Future studies will explore the physiological implications of retinal morphine accumulation and how it might contribute to opioid-induced alterations in both visual functions and circadian regulation of sleep/wake behaviors.

## Data Availability

Publicly available datasets were analyzed in this study. This data can be found here: https://github.com/thebergular/morphine_pk_retina (accessed on 21 April 2022).

## Supplementary Materials

The following supporting information can be downloaded at: https://www.mdpi.com/article/10.3390/ph15050527/s1, Figure S1: Morphine accumulates in the retina following chronic systemic exposure; Figure S2: Hypothalamus and cortex show no differences in morphine concentration per mg tissue; Figure S3: Morphine deposits more in the retina than in the hypothalamus following repeated systemic administration; Figure S4: Morphine accumulates in the retina but not the hypothalamus at ZT2 and ZT9; Table S1: Primer and probe sequences for qRT-PCR experiments; Table S2: Matrix validation results for morphine LC-MS/MS; Table S3: Parameters and acceptance criteria for morphine LC-MS/MS matrix validation.
