# Supplementary material for: Morphine Accumulates in the Retina Following Chronic Systemic Administration
Source: Pharmaceuticals (Basel). 2022 Apr 25;15(5):527. doi: 10.3390/ph15050527 (PMC9146690; doi:10.3390/ph15050527)
Supplement: Supplementary file 1 [file pharmaceuticals-15-00527-s001.zip › pharmaceuticals-1675421-supplementary.pdf]

Supplementary Material

Morphine Accumulates in the Retina Following Chronic Systemic Administration

Nikolas Bergum [1], Casey-Tyler Berezin [2], Gregory Dooley [3] and Jozsef Vigh [1,2,*]

[1] Department of Biomedical Sciences, Colorado State University, Fort Collins, CO 80523, USA; nbergum@colostate.edu
[2] Cell and Molecular Biology Graduate Program, Colorado State University, Fort Collins, CO 80523, USA; ct.berezin@colostate.edu
[3] Department of Environmental and Radiological Health Sciences, Colorado State University, Fort Collins, CO 80523, USA; gregory.dooley@colostate.edu
* Correspondence: jozsef.vigh@colostate.edu; Tel.: +1-970-491-5758

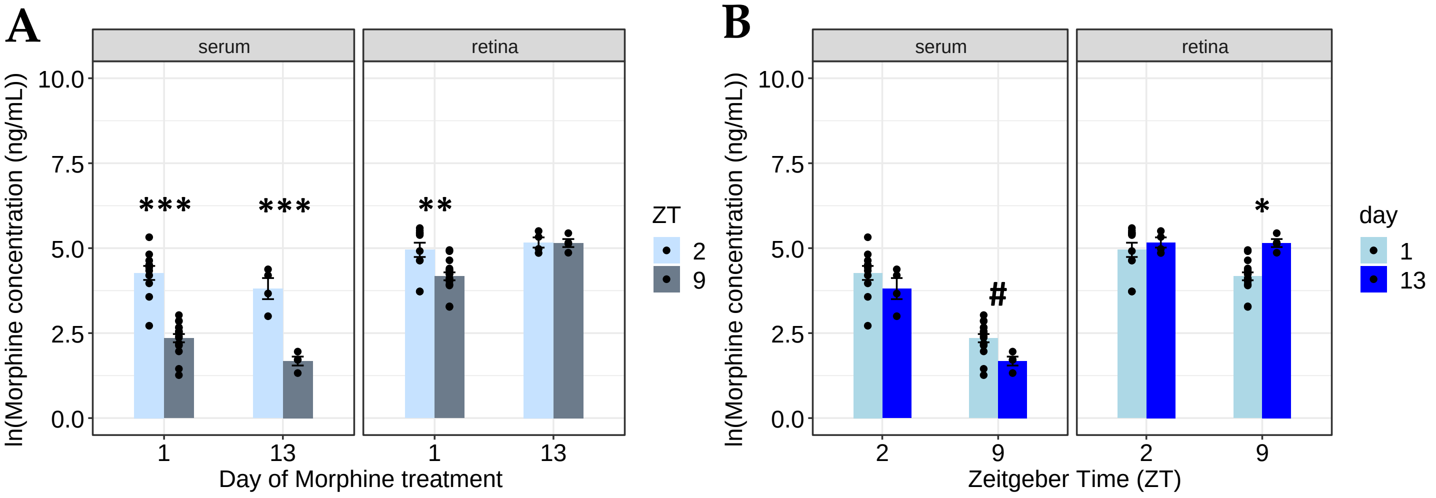

Figure S1. Morphine accumulates in the retina following chronic systemic exposure. (**A, B**) Morphine appears accumulate in the retina following 13 days of chronic morphine treatment, while the serum pharmacokinetics remain similar for samples collected at ZT 2 and ZT 9. Three-Way ANOVA with a Tukey post-hoc adjustment natural logarithmic (ln) scale. (# $p < 0.05$, * $p < 0.001$, ** $p < 0.001$, *** $p < 0.0001$). Data presented as the mean ± SEM.

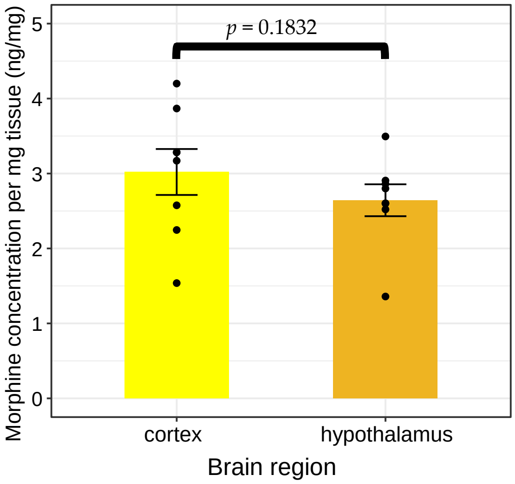

Figure S2. Morphine deposits similarly in different brain regions. Hypothalamus and cerebral cortex samples collected from the same mice show no difference in morphine deposition an hour (ZT 1) after a single 20 mg/kg i.p. morphine injection. Data presented as the mean ± SEM and assessed using a paired samples t-test (n = 8).

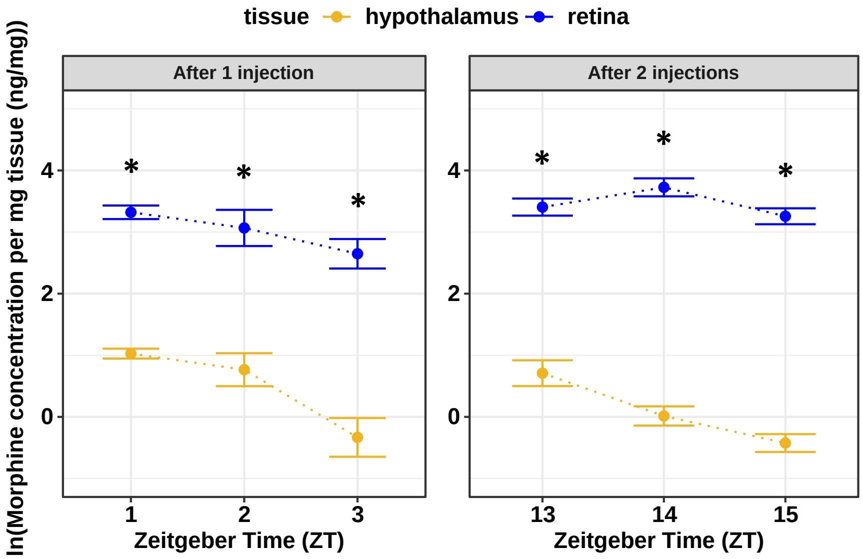

Figure S3. Morphine deposits more in the retina than the hypothalamus following repeat systemic administration**.** Morphine concentration retina and hypothalamus normalized for weight of each tissue after one and two morphine injections. Two-Way ANOVA with a Tukey post-hoc adjustment performed on natural logarithmic (ln) scale. Data presented as the mean ± SEM (* $p < 0.0001$).

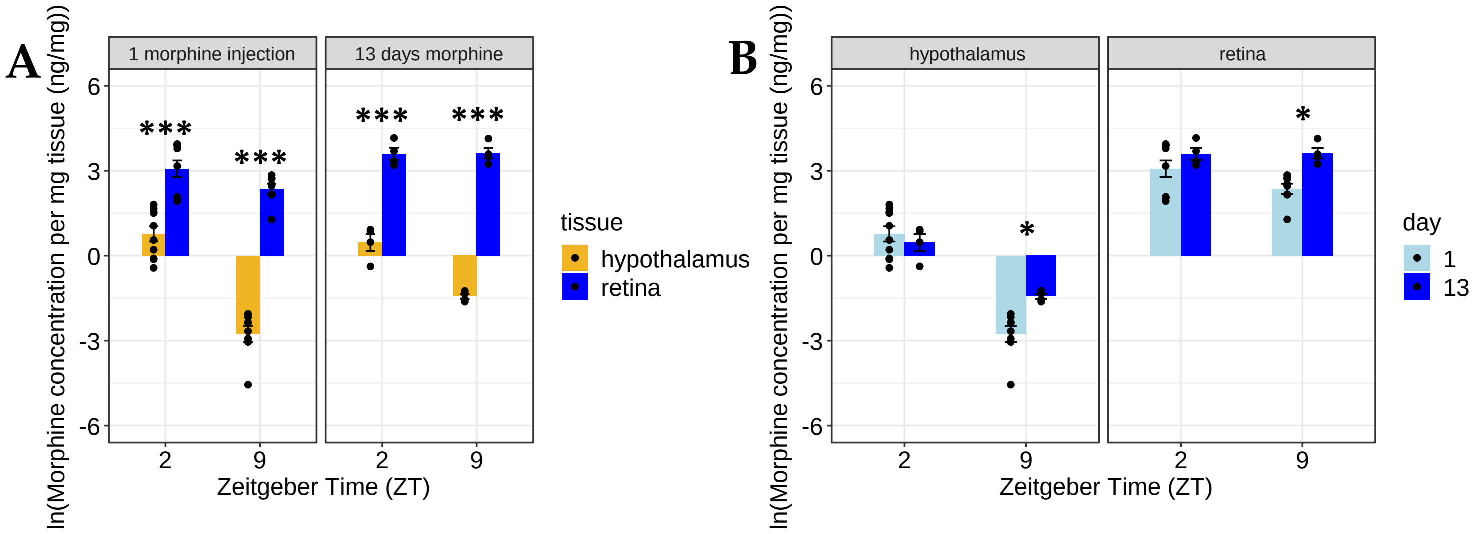

Figure S4. Morphine accumulates in the retina, but not the hypothalamus at ZT 2 and ZT 9**.** (**A**) Morphine concentrations exceed hypothalamic morphine concentrations at ZT 2 and ZT 9 following chronic morphine exposure (13 days morphine). (**B**) Morphine accumulates in the retina, but not the hypothalamus following chronic morphine exposure. Two-Way ANOVA with a Tukey post-hoc adjustment performed on natural logarithmic (ln) scale. Data presented as the mean ± SEM (* p < 0.01, *** *p* < 0.0001).

Table S1. Primer and probe sequences for qRT-PCR experiments.

Target, GenBank accession number	Forward Primer (5’-3’)	Reverse Primer (5’-3’)	Probe Sequence (5’-3’)
P-glycoprotein (Abcb1a), NM_011076.3	CAGCCAGCATTCTCCGT AATA	CCCAAGGATCAGAAACAACA	/FAM/CAGCGGCAG/ZEN/AACA GCAACTTGTTT/IABkFQ/
β-actin (Actb), NM_007393.5	GTCATCCATGGCGAAC TGG	ACTGTCGAGTCGCGTCC	/HEX/CGTTGCCGG/ZEN/ TCCACACCCGCCA/IABkFQ/
TATA box binding protein (Tbp), NM_013684.3	CCATGAAATAGTGATG CTGGGC	GGGTATCTGCTGGCGGTTT	/HEX/TGCGGTCGC/ZEN/ GTCATTTTCTCCGCAGT/IABkFQ/

Table S2. Matrix validation results for morphine LC-MS/MS.

Matrix	Accuracy	Precision	Range ng/ml	Calibration Model	Carryover	Interference	LOQ ng/ml	Stability
Retina	94-98 %	4.6-6.2%	20-5000	Linear	None	None	20	72h
Serum	87-105%	0.84-5.9%	0.5-1000	Linear	None	None	0.5	72h
Hypothalamus	94.2-108%	2.9-13.9%	0.2-50	Linear	None	None	0.2	72h

Table S3. Parameters and acceptance criteria for morphine LC-MS/MS matrix validation.

Parameter	Acceptance Criteria
**Accuracy**	Must not exceed +/- 20% at 3 concentrations
**Precision**	% CV must not exceed 20% at 3 concentrations
**Calibration Model**	Linear Range with R^2 greater then 0.99 (linear mode with 1/x weighting if appropriate fit)
**Carryover**	Carryover from highest calibrator to subsequent blank samples does not exceed 20% of signal of lowest calibrator
**Interference Studies**	No interfering signal form matrix, internal standards, standard, or other common drugs of abuse, OTC drugs, and prescription medications
**Limit of Quantitation (LOQ)**	Lowest concentration that meets accuracy and precision acceptance criteria
**Processed Sample Stability**	Length of time that analyte in extracted sample can be stored at room temperature on autosampler rack and meet accuracy and precision criteria
